# Dentists’ situation and their needs during the COVID-19 pandemic in Nepal: an online questionnaire survey

**DOI:** 10.1186/s12903-022-02139-9

**Published:** 2022-04-01

**Authors:** Yuriko Harada, Hanako Iwashita, Dilip Prajapati, Tomohiko Sugishita

**Affiliations:** 1 International Affairs and Tropical Medicine, Tokyo Women’s Medical University, 8-1 Kawada-chou, Shinjuku-ku, Tokyo, 162-8666 Japan; 2 Department of Community Dentistry, Dhulikhel Hospital, Kathmandu University School of Medical Science, Dhulikhel, Kavrepalanchok, 11008 Nepal

**Keywords:** COVID-19, Pandemic, Dentists, Nepal, Support, Demand-driven

## Abstract

**Background:**

During the coronavirus disease (COVID-19) pandemic, ordinary dental services were sustained in Nepal. Because a dental practice is considered to involve a high risk of infection, the needs of dentists should be identified, and demand-driven support should be provided. The purpose of this study was to investigate the situation and needs of dentists during the COVID-19 pandemic in order to guide demand-driven support. First, we investigated how the situation of Nepali dentists differed according to their types of practices between private clinics and university/government hospitals. Second, we assessed the characteristics of dentists demanding four types of support: financial, material, technical, and guidelines/guidance support.

**Methods:**

A cross-sectional online questionnaire survey was conducted between July 28th and August 7th 2020. Closed-ended questions were prepared regarding behavior, material availability, economic and psychological impacts, training, and the main support dentists wanted to receive. The situation of dentists between private clinics and university/government hospitals was determined using a chi-squared test for each variable. To examine the association between the characteristics of dentists and four types of support, multivariable logistic regression analyses were used to estimate adjusted odds ratios (ORs) with a 95% confidence interval (CI) for each variable.

**Results:**

There were 352 dentists (137 males and 215 females) included in the analysis. Private clinic dentists experienced a bigger economic impact and demanded financial support that 45.5% of them did not receive a salary, compared to 18.9% in university/government hospitals. On the contrary, university/government hospitals experienced lack of PPE and demanded material support that 79.8% had personal protective equipment, compared to 92.5% in private clinics. Financial support was demanded significantly more by male than female dentists (ORs = 5.56; 95% CI = 2.96–10.45). Material support was demanded significantly more by dentists who received training regarding COVID-19 management (ORs = 1.96; 95% CI = 1.01–3.81). Technical support was demanded significantly less by male dentists (ORs = 0.44; 95% CI = 0.23–0.83). Guideline/guidance support was demanded significantly more by dentists who answered that Nepal Dental Association provided appropriate support (ORs = 2.21; 95% CI = 1.25–3.91).

**Conclusion:**

This study articulated the diverse needs of Nepali dentists during the COVID-19 pandemic. Demand-driven support should be provided in the future.

**Supplementary Information:**

The online version contains supplementary material available at 10.1186/s12903-022-02139-9.

## Background

On December 31st 2019, extraordinary pneumonia cases in Wuhan, Hubei Province, China were reported to the World Health Organization (WHO) [1], which was later named coronavirus disease (COVID-19) caused by severe acute respiratory syndrome coronavirus 2 (SARS-CoV-2) [2]. On January 30th 2020, the WHO declared a “Public Health Emergency of International Concern (PHEIC)” due to the catastrophic expansion of COVID-19 worldwide [3]. One of the transmission modes of COVID-19 is airborne transmission, defined as dry respiratory aerosols greater than 5 µm in diameter, from the evaporation of droplets [4, 5]. Transmission could occur while performing aerosol generating procedures (AGP) in routine dental care, such as when using rotary dental and surgical instruments, such as handpieces, ultrasonic scalers, and air–water syringes [6–8]. This increases the risk of infection for dental care professionals and patients, and dental practice is considered to involve a high-risk for cross-infection [7, 9].

Nepal had its first confirmed COVID-19 case on January 23rd 2020 [10], and the number of cases and deaths gradually climbed to 241,995 and 1614, respectively, as of December 8th 2020 [11]. Nepal implemented a nationwide lockdown from 24th March 2020 [12] to restrict movement and community interactions to suppress the spread of infection [13]. On 20th May 2020, the Nepal Dental Association (NDA) published interim guidance for dental practices to protect and prevent COVID-19 infection in dental settings [14]. Nepal decided not to close dental offices but to continue dental services with case prioritization, infection control, and transmission reduction [14]. Although nationwide lockdown measures were lifted on July 22nd 2020 [15], the threat of COVID-19 expansion in Nepal was considered as high [16]. Thus, the risk of cross-infection in dental practices remains high.

In Nepal, almost half of dentists work in private clinics, and the others work in university or government hospitals [17]. A previous systematic review in low- and middle-income countries reported that the private sector more frequently violated medical standards, had poorer patient outcomes, but had better timeliness and hospitality [18]. On the other hand, this systematic review illustrated that the public sector had more limited availability of equipment, medication, and trained healthcare workers [18]. Furthermore, a study from Nepal reported that dentists in university/government hospitals had a better understanding of COVID-19 than dentists in private clinics. [19] Thus, the situation of dentists might have varied based on their types of practices (private clinics or university/government hospitals) in terms of standard precaution practice, material availability, economic and psychological impacts, and training and support dentists wanted to receive during the COVID-19 pandemic. To meet the needs of Nepali dentists efficiently, demand-driven support should be provided. In this study, demand-driven support is defined as support requested by the dentists.

To our knowledge, there has been no study conducted to understand how the situation of Nepali dentists differed based on the types of practices during the COVID-19 pandemic. Additionally, no study has examined the characteristics of dentists for specific types of support, such as financial, material, technical, and guideline/guidance support. In order to provide demand-driven support, the differences in situation of Nepali dentists between private clinics and university/government hospitals and the characteristics of dentists demanding each type of support should be identified. The purpose of this study was to assess the situation and needs of dentists during the COVID-19 pandemic in Nepal in order to guide demand-driven support. There were two aims to achieve this purpose. First, this study aimed to investigate how the situation of Nepali dentists, such as standard precaution practice, material availability, economic and psychological impacts, and training and support, differed based on their types of practices between private clinics and university/government hospitals. Second, we assessed the characteristics of dentists demanding four type of support: financial, material, technical, and guideline/guidance support.

## Methods

### Study design

We conducted a cross-sectional study by distributing an online questionnaire survey among dentists working in Nepal.

### Study population, inclusion and exclusion criteria

The study population consisted of dentists working in Nepal. The inclusion criteria were Nepali dentists working in a clinical setting who consented to participate in the study. Our exclusion criteria were foreign dentists working in Nepal or Nepalese dentists working outside of Nepal.

### Sample size

According to the census conducted in 2015, 1803 people were registered as dentists in Nepal [17]. Among the 1803 dentists, 1318 (73%) lived in Nepal; however, only 1047 (58%) were professionally active [17]. It is estimated that 220 new dentists are registered every year with the Nepal Medical Council [20]. Therefore, the total number of dentists working actively in Nepal in 2020 was estimated as 2147. We did not consider the number of dentists retiring due to a lack of data availability. Based on the estimated population size of 2147, the sample size was calculated using Open-EPI sample size calculator with N = 2147, *p* = 50% ± 5, d = 5%, and 95% confidence interval (CI). [21] This showed that we needed to have minimum 326 participants in the study with 95% CI and 5% margin of error.

### Sampling and data collection

A close-ended online questionnaire was prepared with Google forms in English after reviewing guidance from various international and national organizations such as WHO [6, 9, 22], the Center for Disease Control and Prevention (CDC) [8], American Dental Association (ADA) [23], National Health Service (NHS) [24, 25], and NDA [14]. A draft questionnaire was shared and revised based on the opinions of two dental public health experts and two public health experts, who were familiar with the situation of Nepal. To ensure the validity of the questionnaire items, we piloted the questionnaire with 10 dentists in Nepal. Based on their feedback, some questions were revised for clarification after the pilot test. For the study, we used a convenient sampling strategy and invited dentists from July 28th to August 7th 2020 by disseminating an online questionnaire form along with a participants’ information sheet, which explained the purpose of the questionnaire. All dentists were given time to completed the questionnaire by August 15th 2020. We used emails and social networking services (SNS) such as Facebook, WhatsApp, and Viber with the cooperation of NDA to invite dentists to participate in the study.

### Study instruments

Participants had to choose a single answer from the multiple choices, and all questions had a choice of “refuse to answer”. The main topics covered in the questionnaire were about demographics, precaution practice, material availability, economic and psychological impacts, and training and support. At the end of the questionnaire, dentists were asked to select one main support they wanted to receive from seven choices: financial support, material support, technical support, guideline/guidance, psychological support, other support, or no support needed. Technical support was defined as assistance and provision of knowledge on how to prevent and control the risk of transmission in a dental setting. The actual questionnaire format is attached in the Additional file 1: Table S1.

### Types of practices

Types of practices was classified as “private clinics” and “university/government hospitals”. Dentists who selected “others” were categorized into private clinics because they may work in unlicensed and unregulated clinics, following the definition of the previous systematic review in low- and middle-income countries [18].

### Variable classification

Some variables were further classified: age was dichotomized into “less than 30 years old” and “more than 30 years old”. Highest degree was classified into “Bachelor of Dental Surgery (BDS) level” and “master’s and Doctor of Philosophy (Ph.D.) level”. Personal protective equipment (PPE) and thermometer availability were categorized into “available” and “not available”. The impact of lockdown to the workplace was dichotomized into “permanently closed” and “not permanently closed”. The economic impact on the workplace was categorized into “had a tremendous impact” and “did not have a tremendous impact”. Risk perception of infection in a dental setting was dichotomized to “high risk” and “medium or low risk”. Lastly, training for COVID-19 management in a dental setting was classified into “had training” and “did not have training”.

### Standard precaution practice

A standard precaution practice was measured based on the following seven behavior indicators: use of gloves, hand washing and sanitization, disinfecting dental units, goggles and face shields, extraoral vacuum, measuring the body temperature of patients, and measuring the body temperature of dentists and staff. Dentists who answered that they used/practiced these standard precautions before the COVID-19 pandemic were scored as 2, used/practiced after the COVID-19 pandemic were scored as 1, and do not use/practice were scored as 0. The total score was calculated for each dentist, and it ranged from 0 to 14. Then, the standard precaution practice was dichotomized into two: scores 0–7 as not good standard precaution practice dentists and 8–14 as good standard precaution practice dentists.

### Statistical analysis

The answers to the questionnaire forms were transferred into an Excel sheet and then transferred to STATA. A descriptive analysis was conducted for each question to calculate the frequency and percentage of each answer. To investigate how the situation of dentists differed by types of practices during the COVID-19 pandemic (1st aim of the study), a descriptive analysis was conducted for each question stratified by the types of practices between private clinics and university/government hospitals, after excluding dentists who answered “refuse to answer”. The chi-squared test was conducted for each question to analyze the difference in the proportion of each variable between private clinics and university/government hospitals. Additionally, to examine the characteristic of dentists demanding each type of support (2nd aim of the study), a descriptive analysis was conducted for each question stratified by the main support demanded by dentists (financial, material, technical, guideline/guidance, psychological) after excluding dentists who answered “refuse to answer”. Multivariable logistic regression analyses were conducted to examine the association between the characteristics of dentists and each type of support adjusting for types of practices (private clinics or university/government hospitals). The dependent variable was the main support demanded by dentists, and a separate analysis was conducted for each support (financial, material, technical, guideline/guidance, psychological). For example, the dependent variable for financial support was dichotomized as follows: 0 = not demanding financial support, 1 = demanding financial support as the main support they wanted to receive. The independent variable was the characteristic of dentists taken from each question. The adjusted odds ratios (ORs), 95% CI, and p-value were calculated for each model, and *p* value < 0.05 was considered to be statistically significant. STATA version 14 was used for the statistical analysis.

## Results

There were 354 dentists who were invited to take the questionnaire, and 352 dentists consented to participate and complete the questionnaire form. The number of dentists who completed the questionnaire was bigger than the minimum sample size calculated. This was because of the convenient sampling strategy we used by distributing questionnaires using emails and SNS.

### Demographics

Table 1 summarizes the demographics of the study participants. Among our study participants, about two-third of the sample were aged below 30 years old, female, BDS holders, and worked in private clinics. More than 85% of the study participants worked in urban areas.

**Table 1:** Demographics of the participants

	N = 352
*Age, N (%)*	
≤ 30 years old	237 (67.3)
> 31 years old	114 (32.4)
Refuse to answer	1 (0.3)
*Gender, N (%)*	
Male	137 (38.9)
Female	215 (61.1)
Refuse to answer	0 (0)
*Highest degree, N (%)*	
BDS (Bachelor of Dental Surgery)	251 (71.3)
MDS (Master of Dental Surgery)	86 (24.4)
Other master's level	13 (3.7)
Ph.D. level	1 (0.3)
Refuse to answer	1 (0.3)
*Work location, N (%)*	
Urban	303 (86.1)
Rural	40 (11.4)
Refuse to answer	9 (2.6)
*Types of practices, N (%)*	
Private clinics	200 (56.8)
University hospitals	94 (26.7)
Government hospitals	35 (9.9)
Others	17 (4.8)
Refuse to answer	6 (1.7)

### How the situation differed by the types of practices

#### Demographics

Table 2 shows the results of the questionnaire, stratified by types of practices between private clinics and university/government hospitals. Regarding the demographics of the participants, private clinics had a significantly higher proportion of dentists aged less than 30 years old, compared to university/government hospitals. Also, private clinics had significantly fewer master’s/Ph.D. holders than university/government hospitals.

**Table 2:** Comparison of answer of the questionnaire by the types of practices

	Private clinics (N = 217)^a^	University/government hospitals (N = 129)^a^	*p* values^b^
*Demographics*			
Age, N (%)			
≤ 30 years old	154 (71.3)	78 (60.5)	**0.038**
> 31 years old	62 (28.7)	51 (39.5)	
Gender, N (%)			
Male	84 (38.7)	51 (39.5)	0.879
Female	133 (61.3)	78 (60.5)	
Highest degree, N (%)			
BDS (Bachelor of Dental Surgery)	174 (80.6)	73 (56.6)	**< 0.001**
Master's/Ph.D. level	42 (19.4)	56 (43.4)	
Work location, N (%)			
Urban	191 (89.3)	110 (86.6)	0.464
Rural	23 (10.8)	17 (13.4)	
*Precaution practice*			
Standard precaution practice, N (%)			
Good standard precaution practice	152 (76.0)	93 (77.5)	0.759
Not good standard precaution practice	48 (24.0)	27 (22.5)	
Restriction of aerosol generating procedures, N (%)			
Practiced during the COVID-19 pandemic	124 (59.6)	76 (61.3)	0.763
Did not practice	84 (40.4)	48 (38.7)	
Suspension of non-emergency dental treatment, N (%)			
Suspended non-emergency treatment	188 (88.3)	119 (93.7)	0.101
Did not suspend non-emergency treatment	25 (11.7)	8 (6.3)	
*Material availability*			
Personal protection equipment, N (%)			
Available	196 (92.5)	99 (79.8)	**0.001**
Not available	16 (7.6)	25 (20.2)	
Thermometer, N (%)			
Available	197 (93.8)	109 (89.3)	0.144
Not available	13 (6.2)	13 (10.7)	
*Economic and psychological impacts*			
Impact of lockdown, N (%)			
Permanently closed	37 (17.2)	11 (8.5)	**0.024**
Not permanently closed	178 (82.8)	118 (91.5)	
Impact on salary, N (%)			
Paid full	20 (10.6)	48 (39.3)	**< 0.001**^**b**^
Paid above 80%	2 (1.1)	4 (3.3)	
Paid between 60 and 80%	7 (3.7)	7 (5.7)	
Paid 40–60%	35 (18.5)	23 (18.9)	
Paid 20–40%	23 (12.2)	10 (8.2)	
Paid below 20%	16 (8.5)	7 (5.7)	
Did not receive any salary	86 (45.5)	23 (18.9)	
Economic Impact on clinic, N (%)			
Had a tremendous impact	45 (22.2)	25 (22.7)	0.910
Did not have a tremendous impact	158 (77.8)	85 (77.3)	
Risk perception of infection in a dental setting, N (%)			
High risk	185 (86.5)	114 (89.8)	0.368
Low/medium risk	29 (13.6)	13 (10.2)	
Impact on psychology, N (%)			
Felt stressed or anxious	195 (92.4)	110 (87.3)	0.121
Did not feel stressed or anxious	16 (7.6)	16 (12.7)	
*Training and support*			
Training for COVID-19 management in a dental setting, N (%)			
Had training	176 (81.1)	100 (77.5)	0.422
Did not have any training	41 (18.9)	29 (22.5)	
Perception of Nepal government, N (%)			
Had appropriate support	1 (0.5)	4 (3.3)	**0.043**
Did not have appropriate support	208 (99.5)	117 (96.7)	
Perception of Nepal Dental Association, N (%)			
Had appropriate support	42 (21.5)	23 (20.2)	0.777
Did not have appropriate support	153 (78.5)	91 (79.8)	
Support dentists wanted to receive, N (%)			
Financial support	42 (20.7)	16 (12.5)	**0.005**^**b**^
Material support	49 (24.1)	46 (35.9)	
Technical support	47 (23.2)	14 (10.9)	
Guideline/guidance	59 (29.1)	46 (35.9)	
Psychological support	6 (3.0)	6 (4.7)	

^a^The total number of dentists in each question may not have been 217 for private clinics and 129 for university/government hospitals because the analysis excluded those who answered “refuse to answer”. The *p* values below 0.05 are shown in bold

^b^A Bonferroni correction was performed for the chi-squared tests to correct for multiple testing

#### Precaution practice

The descriptive analysis showed that 345 (98.0%) of dentists answered that they changed gloves for each patient before the pandemic. Also, 300 (85.2%) dentists answered they washed/sanitized their hands before the COVID-19 pandemic, and the rest of the dentists started to practice this after the COVID-19 pandemic. On the contrary, the use of dental goggles and face shields was not common before the COVID-19 pandemic: only 47 (13.4%) practiced their use before the COVID-19 pandemic; however, 291 (82.7%) started to use goggles and face shields after the COVID-19 pandemic. Additionally, 258 (73.3%) and 292 (83.0%) dentists started to measure dentists’ and patients’ body temperature after the COVID-19 pandemic, respectively. As shown in Table 2, more than 75% of dentists in both private clinics and university/government hospitals had good standard precaution practice. Our analysis did not find significant differences of standard precaution practice, restriction of AGP, and suspension of non-emergency treatment between private clinics and university/government hospitals.

#### Material availability

As Table 2 shows, materials were less available in university/government hospitals: the proportion of dentists who answered that PPE was not available was more than double in university/government hospitals, compared to private clinics.

#### Economic and psychological impacts

Private clinic dentists faced a bigger economic impact as described in Table 2: around 17% of clinics were permanently closed, which was about double, compared to university/government hospitals. Also, around half of private clinic dentist did not received any salary, which was more than double than university/government hospital dentists. The majority of dentists considered that dental settings were a high risk for infection transmission.

#### Training and support

There was no significant difference in training for COVID-19 management in a dental setting between private clinics and university/government hospitals. Among 176 and 100 dentists who received training, 86 (48.9%) and 26 (26.0%) dentists received training on social media for private clinics and university/government hospitals, respectively. As Table 2 shows, a higher proportion of dentists in private clinics demanded financial and technical support than university/government hospitals. On the contrary, a higher proportion of dentists in university/government hospitals demanded material, guideline/guidance, and psychological support, compared to private clinic dentists. There was no dentist who chose “other support” or “no support needed”.

### Dentists demanding each type of support

Additional file 2: Table S2 describes the results of the questionnaire stratified by the main types of support demanded by dentists (financial, material, technical, and guideline/guidance support), adjusted by types of practices. Table 3 shows the results of multivariate logistic regression analyses. Analysis of psychological support was not conducted due to the limited sample number (n = 12).

**Table 3:** Multivariate logistic regression analyses for the answer of the questionnaire and main support dentists demand

	Financial support (n = 58)^a)^			Material support (n = 95)^a^			Technical support (n = 61)^a^			Guideline/guidance (n = 105)^a^		
	ORs	95% CI	*p* values	ORs	95% CI	*p* values	ORs	95% CI	*p* values	ORs	95% CI	*p* values
*Demographics of participants*												
Age												
30 years old	0.41	0.23–0.74	**0.003**	2.10	1.20–3.65	**0.009**	1.21	0.65–2.25	0.551	1.05	0.64–1.72	0.856
> 31 years old	Ref			Ref			Ref			Ref		
Gender												
Male	5.56	2.96–10.45	**< 0.001**	1.04	0.64–1.70	0.867	0.44	0.23–0.83	**0.011**	0.51	0.31–0.83	**0.007**
Female	Ref			Ref			Ref			Ref		
Highest degree												
BDS (bachelor of dental surgery)	Ref			Ref			Ref			Ref		
Master’s/Ph.D. level	2.35	1.26–4.38	**0.008**	0.60	0.34–1.06	0.081	0.69	0.34–1.39	0.299	1.07	0.63–1.81	0.805
Work location												
Urban	1.11	0.44–2.81	0.819	1.14	0.53–2.46	0.738	0.93	0.39–2.24	0.872	1.04	0.50–2.15	0.918
Rural	Ref			Ref			Ref			Ref		
*Behavior*												
Precaution practice score												
Good precaution practice	1.31	0.62–2.77	0.481	0.86	0.48–1.54	0.622	1.22	0.60–2.47	0.578	1.18	0.66–2.10	0.573
Not good precaution practice	Ref			Ref			Ref			Ref		
Restriction of aerosol generating procedures												
Practiced during the Covid-19 pandemic	1.50	0.81–2.76	0.197	0.77	0.47–1.27	0.306	0.60	0.34–1.06	0.077	1.25	0.77–2.03	0.377
Did not practice	Ref			Ref			Ref			Ref		
Suspension of non-emergency dental treatment												
Suspended non-emergency treatment	0.46	0.20–1.03	0.058	0.93	0.41–2.10	0.861	1.76	0.59–5.27	0.310	1.34	0.58–3.10	0.491
Did not suspend non-emergency treatment	Ref			Ref			Ref			Ref		
*Material availability*												
Personal protection equipment												
Available	1.28	0.47–3.48	0.630	0.49	0.25–0.98	**0.044**	1.09	0.43–2.78	0.856	1.89	0.85–4.18	0.118
Not available	Ref			Ref			Ref			Ref		
Thermometer												
Available	5.34	0.71–40.40	0.104	0.49	0.21–1.12	0.090	5.66	0.75–42.85	0.093	0.60	0.26–1.37	0.223
Not available	Ref			Ref			Ref			Ref		
*Economic and psychological impacts*												
Impact of lockdown												
Permanently closed	0.92	0.40–2.10	0.841	0.73	0.35–1.55	0.415	1.11	0.51–2.39	0.797	1.10	0.57–2.15	0.775
Not permanently closed	Ref			Ref			Ref			Ref		
Impact on salary												
Paid full	0.24	0.08–0.77	**0.017**	1.21	0.58–2.53	0.611	0.24	0.08–0.77	**0.017**	1.45	0.71–2.96	0.302
Paid above 80%	1.91	0.32–11.39	0.478	1.28	0.21–7.61	0.790	1.91	0.32–11.39	0.478	1.24	0.21–7.31	0.811
Paid between 60 and 80%	0.60	0.13–2.92	0.531	1.57	0.47–5.23	0.462	0.60	0.13–2.92	0.531	1.46	0.44–4.79	0.534
Paid 40–60%	0.74	0.32–1.68	0.464	1.35	0.65–2.79	0.418	0.74	0.32–1.68	0.464	1.13	0.55–2.31	0.747
Paid 20–40%	1.29	0.53–3.15	0.572	1.01	0.40–2.55	0.978	1.29	0.53–3.15	0.572	0.62	0.23–1.67	0.345
Paid below 20%	0.72	0.23–2.33	0.590	1.12	0.39–3.17	0.834	0.72	0.23–2.33	0.590	1.50	0.57–3.92	0.412
Did not receive any salary	Ref			Ref			Ref			Ref		
Economic Impact on clinic												
Had a tremendous impact	5.51	2.93–10.38	**< 0.001**	0.61	0.32–1.17	0.135	0.35	0.14–0.85	**0.020**	0.55	0.29–1.03	0.062
Did not have a tremendous impact	Ref			Ref			Ref			Ref		
Risk perception of infection in a dental setting												
High risk	1.05	0.44–2.51	0.914	1.06	0.51–2.22	0.878	1.16	0.48–2.77	0.744	0.82	0.41–1.63	0.563
Low/medium risk	Ref			Ref			Ref			Ref		
Impact on psychology												
Felt stressed or anxious	0.60	0.24–1.50	0.276	0.85	0.38–1.91	0.696	3.06	0.70–13.30	0.137	0.99	0.45–2.19	0.977
Did not feel stressed or anxious	Ref			Ref			Ref			Ref		
*Training and support*												
Training for Covid-19 management in a dental setting												
Had training	1.08	0.53–2.23	0.827	1.96	1.01–3.81	**0.046**	1.01	0.50–2.05	0.969	1.15	0.64–2.05	0.643
Did not have any training	Ref			Ref			Ref			Ref		
Perception of Nepali government												
Had appropriate support	4.18	0.65–26.67	0.131	N/A	N/A	N/A	1.71	0.18–16.45	0.640	1.40	0.23–8.63	0.718
Did not have appropriate support	Ref			Ref			Ref			Ref		
Perception of Nepal Dental Association												
Had appropriate support	0.58	0.26–1.29	0.179	0.65	0.34–1.26	0.204	1.33	0.67–2.65	0.413	2.21	1.25–3.91	**0.006**
Did not have appropriate support	Ref			Ref			Ref			Ref		

*OR* adjusted odds ratio, *CI* confidence interval, *Ref* reference

^a^The total number of dentists in each question may not have been 58 for financial, 95 for material, 61 for technical, and 105 for guideline/guidance because the analysis excluded those who answered “refuse to answer”. All analyses were adjusted for type of practice. The *p* values below 0.05 are shown in bold

#### Financial support

As Table 3 shows, a tendency was observed that financial support was demanded more by male dentists (ORs = 5.56; 95% CI = 2.96–10.45). Additionally, dentists with master’s/Ph.D. holders, and who experienced a tremendous economic impact had significantly higher ORs for demanding financial support. On the contrary, dentists aged less than 30 years old, and were paid a full salary had significantly lower adjusted ORs for demanding financial support.

#### Material support

As Table 3 shows, material support was demanded significantly more by dentists who received training regarding COVID-19 management (ORs = 1.96; 95% CI = 1.01–3.81). Additionally, male dentists had significantly higher ORs for demanding material support. On the contrary, dentists who answered that PPE was available had significantly lower ORs for demanding material support.

#### Technical support

As Table 3 shows, technical support was demanded significantly less by male dentists. (ORs = 0.44; 95% CI = 0.23–0.83). Also, dentists who were paid a full salary and who experienced a tremendous economic impact had significantly lower ORs for demanding technical support.

#### Guideline/guidance support

As Table 3 shows, dentists who answered that NDA provided appropriate support had significantly higher ORs for demanding guidelines/guidance (ORs = 2.21; 95% CI = 1.25–3.91). On the contrary, males had significantly lower ORs for demanding guidelines/guidance.

## Discussion

The purpose of this study was to assess the situation and need of dentists during the COVID-19 pandemic in order to provide demand-driven support. Dentists working in private clinics experienced a more severe economic impact and demanded more for financial and technical support. On the contrary, dentists in university/government hospital dentists experienced a lack of PPE and demanded more for material and guideline/guidance support. Additionally, there were diverse needs of Nepali dentists demanding financial, material, technical, and guideline/guidance support even after adjusting for types of clinics. Financial support was demanded significantly more by male dentists and dentists with a bigger economic impact. Material support was significantly demanded who experienced a lack of PPE. Technical support was demanded significantly less by male dentists. Lastly, guideline/guidance was especially demanded by dentists who responded that NDA gave the appropriate support.

This study examined that dentists in Nepal had a better adherence to the standard precautions practice, compared to studies reported from other countries during the COVID-19 pandemic [26–29]. For example, all dentists in our study reported that they washed their hands for each patient during the COVID-19 pandemic, and this was also higher than studies in Italy (91.6%), Jordan (96.2%), and a multi-country study (94.0%) [27–29]. The reason for the better adherence to the standard precaution practice in our study among Nepali dentists than studies from other countries was not clear, but this could be due to the different risk perception of infection in dental settings: our study showed that more than 85% of dentists in Nepal perceived dental settings as a high risk of infection, and this was higher than a study in Italy (64.5%) [27]. Additionally, our study found that standard precaution practice was not significantly different between private clinics and university/government hospitals. This tendency was different from a previous systematic review on low- and middle-income countries in which private care providers tended to violate medical standards by not following the treatment protocol and guidelines [18]. Further study should be conducted whether this tendency remains even after the COVID-19 pandemic.

Our study showed that dentists working in private clinics experienced a larger economic impact and demanded more financial support: a higher proportion of dentists responded that their clinics were permanently closed and did not receive any salary, compared to university/government hospitals. This tendency was aligned with a previous study that reported a significantly higher percentage of dental practice closure in the private sector compared to non-private sector during the COVID-19 pandemic [30]. Some countries announced a financial support scheme for dentists such as the United Kingdom [31], United States [32], and Japan [33]. However, as of October 16^th^ 2021, there is no financial support scheme available for dentists in Nepal. Financial support should be provided to dentists in Nepal especially in the private sector.

Additionally, dentists working in private clinics demanded more technical support. This could be partly explained by a difference of the training dentists received regarding COVID-19 management between private clinics and university/government hospitals. Although our analysis did not find any difference in whether dentist received training or not, the source of training was different: almost 50% in private clinics and 25% in university/government hospitals had training by social media. Social media plays an important role in changing public attitudes, behavior, and awareness especially during a pandemic situation [34]; however, there is some fake and misleading information [35, 36]. Because a dental setting is considered a high-risk for nosocomial infection [9], dentists should be able to get access to evidence-based information and training sources for COVID-19 prevention and management, especially for private clinics dentists.

On the contrary, a higher proportion of dentists working in university/government hospitals reported a lack of PPE and demanding for material support. This tendency also coincided with a previous a systematic review of low- and middle-income countries for which the public sector experienced a limited availability of equipment [18]. As suggested by WHO, use of PPE was crucial during the COVID-19 pandemic to protect dentists and patients from infection [22]. This calls for urgent action to provide needed equipment for dentists to ensure that PPE is available, especially among university/government hospitals.

Moreover, our study examined the characteristics of dentists demanding financial, material, technical, and guideline/guidance support after adjusting for types of practices. Financial support was significantly demanded by male dentists. The reason why male dentists demanded financial support was not clear; however, one study in Spain investigated that the economic impact during the COVID-19 pandemic was bigger among male than female dentists. [37] Future studies should address why more male dentists demand financial support than female dentists. Material support was significantly demanded by dentists who received training regarding COVID-19 management. Through a training regarding COVID-19 management, dentists might be able to understand the importance of using PPE. This might explain the reason why material supports were significantly demanded by dentists who received COVID-19 management training. Technical support was demanded significantly less by male dentists. This could be explained by a previous study in Nepal that male dentists had better knowledge about COVID-19 than female dentists [19]. With a better knowledge about COVID-19 among male dentists, they might understand how to minimize the cross-infection at dental practice thus demanded less for the technical supports. Guidelines/guidance was especially demanded by dentists who responded that NDA gave appropriate support. After the declaration of PHEIC, it took almost four months until WHO published its guidance for dental settings on 1st June 2020. However, NDA took a faster initiative to publish its guidance on 20th May 2020 [14]. Therefore, guidance for Nepali dentists was available from NDA and from WHO while this study was conducted between July 28th and August 7th 2020. Further study is needed about whether dentists in Nepal are aware of these guidelines and what aspect of guidelines/guidance they wanted to have.

This study has implications for the significance of demand-driven support for Nepali dentists during the COVID-19 pandemic. Our analysis articulated the differences in the situation and needs of dentists based on the types of practices between private clinics and university/government hospitals. Moreover, there were diverse needs of dentists for financial, material, technical, and guideline/guidance support even after adjusting for types of practices. Various countries and organizations have announced to support Nepal to minimize the impact of the Covid-19 pandemic [38]. The appropriate support should be given to Nepali dentists, and the national government and international partners should consider types of practices and the different characteristics of dentists demanding suggested by this study to provide demand-driven support. Further researches and stakeholder engagement are needed to better inform policymaking and donor decisions to reflect the needs of the dentists. Another important implication is that our study showed that some standard precautionary practices were adopted after the COVID-19 pandemic. For example, 13.4% of dentists used goggles and face shields before the COVID-19 pandemic, and 82.7% of dentists started to practice this after the COVID-19 pandemic. This practice should continue to be encouraged even after the COVID-19 pandemic, and this pandemic is an important opportunity for dentists to include these standard precautionary practices into their routine practice. Because this study was conducted at an early stage of the COVID-19 pandemic, a follow-up study is needed to investigate how the standard precautionary practices, situation and needs of dentists have changed.

This study had several limitations. First, we used a convenience sampling strategy by disseminating questionnaire forms by email and SNS, which could have resulted in selection bias and lack of generalizability. It was not possible to conduct random sampling because there was no registry of dentists available. However, our study illustrated that 61.1% of the study sample were female, which was similar to a previous national census data of 59.4%, conducted in 2015 [17]. Second, this study was a questionnaire-based study so the answers may have been prone to response bias. Moreover, we did not use the standardized questionnaire due to the absence of appropriate questionnaire forms which meet our study purpose. The answer of the questionnaire was a single choice. Dentists who are equally in needs of multiple support had to select a single support they wanted to receive. Also, dentists who selected “others” (n = 17) for their workplace was categorized as private clinic dentists. Thus, the results may have validation and reliability issues and should be carefully interpreted. However, the analysis results supported the consistency of our data. For example, financial support was demanded by dentists who experienced a big economic impact, and material support was demanded by dentists who faced lack of material availability. Thus, the issue of validity and reliability might have been small.

## Conclusion

This study assessed the needs of dentists in Nepal to guide demand-driven support during the COVID-19 pandemic. Our analysis showed that the situation of Nepali dentists during the COVID-19 pandemic differed according to the types of practices between private clinics and university/government hospitals. Additionally, there were diverse needs of Nepali dentists for financial, material, technical, and guideline/guidance support even after adjusting for type of practices. Our study articulated the significance of the demand-driven support by the national government and international partners to Nepali dentists, and consideration should be given based on types of practices between private clinics and university/government hospitals and other characteristics suggested by this study.

## Supplementary Information

**Additional file 1: Table S1.** Questionnaire.

**Additional file 2: Table S2.** Comparison of answer of the questionnaire by the main support dentists demand.

## Data Availability

The datasets used and analyzed during the current study are available from the corresponding author on reasonable request.

## Abbreviations

ADA: American Dental Association
AGP: Aerosol generating procedures
BDS: Bachelor of Dental Surgery
CDC: Center for Disease Control and Prevention
CI: Confidence interval
COVID-19: Coronavirus disease
MDS: Master of Dental Surgery
NDA: Nepal Dental Association
NHS: National Health Service
ORs: Odds ratios
Ph.D.: Doctor of Philosophy
PHEIC: Public Health Emergency of International Concern
SARS-CoV-2: Severe acute respiratory syndrome coronavirus 2
SNS: Social networking services
WHO: World Health Organization
