# Supplementary material for: Dentists’ situation and their needs during the COVID-19 pandemic in Nepal: an online questionnaire survey
Source: BMC Oral Health. 2022 Apr 1;22:107. doi: 10.1186/s12903-022-02139-9 (PMC8973669; doi:10.1186/s12903-022-02139-9)
Supplement: Supplementary file 1 — Additional file 1: Table S1. Questionnaire. [file 12903_2022_2139_MOESM1_ESM.docx]

**Additional file 1**

**Dentists’ situation and their needs during the COVID-19 pandemic in Nepal: An online questionnaire survey**

Yuriko Harada^1^, Hanako Iwashita^1^, Dilip Prajapati^2^, Tomohiko Sugishita^1^

1. International Affairs and Tropical Medicine, Tokyo Women's Medical University, 162-8666, 8-1 Kawada-chou, Shinjuku-ku, Tokyo, Japan
2. Community and Public Health Dentistry, Dhulikhel Hospital, Kathmandu University School of Medical Science, 11008, Dhulikhel, Kavrepalanchok, Nepal

**Corresponding author:** Yuriko Harada, yurikoharada22@gmail.com

| **Supplementary Table S1. Questionnaire** | |
| --- | --- |
| **Demographics** | |
| What is your age? | ( ) years old |
| What is your gender? | Male |
|  | Female |
|  | Refuse to answer |
| What is your highest degree? | BDS (bachelor of dental surgery) |
|  | MDS (master of dental surgery) |
|  | Other master's level |
|  | Ph.D. |
|  | Refuse to answer |
| Where do you work as a dentist? | Rural |
|  | Urban |
|  | Refuse to answer |
| Where do you work as a dentist at most? | Private clinics |
|  | University hospitals |
|  | Government hospital |
|  | Others |
|  | Refuse to answer |
| **Precaution practice** |  |
| Choose the most appropriate statement regarding "changing gloves for each patient". | Practice before the COVID-19 pandemic |
|  | Practice since the COVID-19 pandemic |
|  | Do not practice |
|  | Refuse to answer |
| Choose the most appropriate statement regarding "Wash and sanitize your hands for each patient". | Practice before the COVID-19 pandemic |
|  | Practice since the COVID-19 pandemic |
|  | Do not practice |
|  | Refuse to answer |
| Choose the most appropriate statement regarding "Cleaning and disinfecting dental units for each patient". | Practice before the COVID-19 pandemic |
|  | Practice since the COVID-19 pandemic |
|  | Do not practice |
|  | Refuse to answer |
| Choose the most appropriate statement regarding "Use dental goggles or face shields". | Practice before the COVID-19 pandemic |
|  | Practice since the COVID-19 pandemic |
|  | Do not practice |
|  | Refuse to answer |
| Choose the most appropriate statement regarding "Use extraoral vacuum". | Practice before the COVID-19 pandemic |
|  | Practice since the COVID-19 pandemic |
|  | Do not practice |
|  | Refuse to answer |
| Choose the most appropriate statement regarding "Measure body temperature of dentists and dental staffs every day before working". | Practice before the COVID-19 pandemic |
|  | Practice since the COVID-19 pandemic |
|  | Do not practice |
|  | Refuse to answer |
| Choose the most appropriate statement regarding "Measure body temperature of patients before treatment". | Practice before the COVID-19 pandemic |
|  | Practice since the COVID-19 pandemic |
|  | Do not practice |
|  | Refuse to answer |
| Did you manage to restrict aerosol generating procedures in your practice?　(eg: treatment using dental air turbine) | Practice since the COVID-19 pandemic |
|  | Do not practice |
|  | Refuse to answer |
| Did you suspend non-emergency dental procedures during the COVID-19 pandemic? | Practice since the COVID-19 pandemic |
|  | Do not practice |
|  | Refuse to answer |
| **Material availability** |  |
| Are Personal Protection Equipment (PPE) and protection gears easily available? | Available with affordable price |
|  | Available but not affordable |
|  | Not available |
|  | Refuse to answer |
| Are body thermometers easily available? | Available with affordable price |
|  | Available but not affordable |
|  | Not available |
|  | Refuse to answer |
| **Economic and psychological impacts** |  |
| What impact does your workplace have through the COVID-19 lockdown? | Permanently closed |
|  | Temporarily closed (Decrease the opening days) during the lockdown period |
|  | Decrease working days or hours during the lockdown period |
|  | Open with normal working days and hours during the lockdown period |
|  | Refuse to answer |
| What impact does your salary have during the COVID-19 crisis? | Paid full |
|  | Paid above 80% |
|  | Paid between 60−80% |
|  | Paid between 40−60% |
|  | Paid below 20% |
|  | Did not receive any salary |
|  | Refuse to answer |
| What is an economic impact on your dental clinics have due to the COVID-19 pandemic? | Had a tremendous economic impact and may lead to bankrupt |
|  | Had some economic impact but possible to reconstruct |
|  | Does not have any economic impact |
|  | Refuse to answer |
| What do you think about the risk of nosocomial infection in dental settings? | Low risk |
|  | Medium risk |
|  | High risk |
|  | Refuse to answer |
| How do you feel about working as a dentist during the COVID-19 pandemic? | Felt stressed or anxious |
|  | Did not feel stressed or anxious |
|  | Refuse to answer |
| **Training and support** |  |
| Did you receive any training on the prevention and control of COVID-19 in dental practice? | Yes at my dental clinic or institution |
|  | Yes at webinar |
|  | Yes at social media |
|  | No |
|  | Refuse to answer |
| What do you think about support by government of Nepal during the COVID-19 pandemic for dentists? | Had appropriate supports |
|  | Did not have appropriate supports |
|  | Refuse to answer |
| What do you think about support by Nepal Dental Association during the COVID-19 pandemic for dentists? | Had appropriate supports |
|  | Did not have appropriate supports |
|  | Refuse to answer |
| What kind of supports do you need the most as a dentist during the COVID-19 pandemic? | Financial support |
|  | Material support |
|  | Technical support (knowledge regarding how to prevent and control the risk of infection) |
|  | Guideline and guidance regarding dental practice during the COVID-19 |
|  | Psychological support |
|  | Others |
|  | No supports needed |
|  | Refuse to answer |
