# Supplementary material for: Dentists’ situation and their needs during the COVID-19 pandemic in Nepal: an online questionnaire survey
Source: BMC Oral Health. 2022 Apr 1;22:107. doi: 10.1186/s12903-022-02139-9 (PMC8973669; doi:10.1186/s12903-022-02139-9)
Supplement: Supplementary file 2 — Additional file 2: Table S2. Comparison of answer of the questionnaire by the main support dentists demand. [file 12903_2022_2139_MOESM2_ESM.docx]

**Additional file 2**

**Dentists’ situation and their needs during the COVID-19 pandemic in Nepal: An online questionnaire survey**

Yuriko Harada^1^, Hanako Iwashita^1^, Dilip Prajapati^2^, Tomohiko Sugishita^1^

1. International Affairs and Tropical Medicine, Tokyo Women's Medical University, 162-8666, 8-1 Kawada-chou, Shinjuku-ku, Tokyo, Japan
2. Community and Public Health Dentistry, Dhulikhel Hospital, Kathmandu University School of Medical Science, 11008, Dhulikhel, Kavrepalanchok, Nepal

**Corresponding author:** Yuriko Harada, yurikoha**r**ada22@gmail.com

**Supplementary Table S2. Comparison of answer of the questionnaire by the main support dentists demand**

|  |  | Financial support  (N=58)^1)^ | Material support  (N =95) ^1)^ | Technical support  (N =61) ^1)^ | Guideline/guidance  (N =105) ^1)^ |
| --- | --- | --- | --- | --- | --- |
| **Demographics** | |  |  |  |  |
| Types of practices, N (%) | |  |  |  |  |
|  | Private clinics | 42 (72.4) | 49 (51.6) | 47 (77.1) | 59 (56.2) |
|  | University/government hospital | 16 (27.6) | 46 (48.4) | 14 (23.0) | 46 (43.8) |
| Age, N (%) | |  |  |  |  |
|  | 30 years old | 30 (51.7) | 73 (76.8) | 44 (72.1) | 70 (67.3) |
|  | >31 years old | 28 (48.3) | 22 (23.2) | 17 (27.9) | 34 (32.7) |
| Gender, N (%) | |  |  |  |  |
|  | Male | 42 (72.4) | 38 (40.0) | 15 (24.6) | 30 (28.6) |
|  | Female | 16 (27.6) | 57 (60.0) | 46 (75.4) | 75 (71.4) |
| Highest degree | |  |  |  |  |
|  | BDS (bachelor of dental surgery) | 35 (60.3) | 72 (75.8) | 49 (80.3) | 72 (69.2) |
|  | Master's/Ph.D. level | 23 (39.7) | 23 (24.2) | 12 (19.7) | 32 (30.8) |
| Work location, N (%) | |  |  |  |  |
|  | Urban | 52 (89.7) | 82 (89.1) | 53 (88.3) | 93 (88.6) |
|  | Rural | 6 (10.3) | 10 (10.9) | 7 (11.7) | 12 (11.4) |
| **Precaution practice** | |  |  |  |  |
| Standard precaution practice, N (%) | |  |  |  |  |
|  | Good standard precaution practice | 41 (80.4) | 66 (75.0) | 46 (79.3) | 78 (78.8) |
|  | Not standard good precaution practice | 10 (19.6) | 23 (25.0) | 12 (20.7) | 21 (21.2) |
| Restriction of aerosol generating procedures, N (%) | | |  |  |  |
|  | Practiced during the COVID-19 pandemic | 38 (67.9) | 51 (56.0) | 30 (50.0) | 64 (64.0) |
|  | Did not practice | 18 (32.1) | 40 (44.0) | 30 (50.0) | 36 (36.0) |
| Suspension of non-emergency dental treatment, N (%) | | |  |  |  |
|  | Suspended non-emergency treatment | 48 (82.8) | 85 (90.4) | 54 (93.1) | 95 (92.2) |
|  | Did not suspend non-emergency treatment | 10 (17.2) | 9 (9.6) | 4 (6.9) | 8 (7.8) |
| **Material availability** | |  |  |  |  |
| Personal protection equipment, N (%) | |  |  |  |  |
|  | Available | 51 (91.1) | 73 (80.2) | 55 (90.2) | 94 (91.3) |
|  | Not available | 5 (8.9) | 18 (19.8) | 6 (9.8) | 9 (8.7) |
| Thermometer, N (%) | |  |  |  |  |
|  | Available | 56 (98.3) | 77 (87.5) | 60 (98.4) | 89 (89.0) |
|  | Not available | 1 (1.8) | 11 (12.5) | 1 (1.6) | 11 (11.0) |
| **Economic and psychological impacts** | |  |  |  |  |
| Impact of lockdown, N (%) | |  |  |  |  |
|  | Permanently closed | 8 (14.0) | 10 (10.6) | 10 (16.4) | 15 (14.3) |
|  | Not permanently closed | 49 (86.0) | 84 (89.4) | 51 (83.6) | 90 (85.7) |
| Impact on salary, N (%) | |  |  |  |  |
|  | Paid full | 4 (7.1) | 22 (25.6) | 9 (16.4) | 25 (27.5) |
|  | Paid above 80% | 2 (3.6) | 2 (2.3) | 0 (0) | 2 (2.2) |
|  | Paid between 60−80% | 2 (3.6) | 5 (5.8) | 2 (3.6) | 5 (5.5) |
|  | Paid 40−60% | 10 (17.9) | 18 (20.9) | 11 (20.0) | 17 (18.7) |
|  | Paid 20%−40% | 9 (16.1) | 8 (9.3) | 9 (16.4) | 6 (6.6) |
|  | Paid below 20% | 4 (7.1) | 6 (7.0) | 2 (3.6) | 8 (8.8) |
|  | Did not receive any salary | 25 (44.6) | 25 (29.1) | 22 (40.0) | 28 (30.8) |
| Economic Impact on clinic, N (%) | |  |  |  |  |
|  | Had a tremendous impact | 28 (50.9) | 14 (16.7) | 6 (10.5) | 15 (15.8) |
|  | Did not have a tremendous impact | 27 (49.1) | 70 (83.3) | 51 (89.5) | 80 (84.2) |
| Risk perception of infection in a dental setting, N (%) | | |  |  |  |
|  | High risk | 50 (87.7) | 84 (88.4) | 53 (88.3) | 88 (86.3) |
|  | Low/medium risk | 7 (12.3) | 11 (11.6) | 7 (11.7) | 14 (13.7) |
| Impact on psychology, N (%) | |  |  |  |  |
|  | Felt stressed or anxious | 49 (87.5) | 82 (89.1) | 58 (96.7) | 94 (90.4) |
|  | Did not feel stressed or anxious | 7 (12.5) | 10 (10.9) | 2 (3.3) | 10 (9.6) |
| **Training and support** | |  |  |  |  |
| Training for COVID-19 management in a dental setting, N (%) | | |  |  |  |
|  | Had training | 47 (81.0) | 82 (86.3) | 49 (80.3) | 85 (81.0) |
|  | Did not have any training | 11 (19.0) | 13 (13.7) | 12 (19.7) | 20 (19.1) |
| Perception of Nepal government, N (%) | |  |  |  |  |
|  | Had appropriate support | 2 (3.5) | 0 (0) | 1 (1.7) | 2 (2.1) |
|  | Did not have appropriate support | 56 (96.6) | 92 (100) | 58 (98.3) | 95 (97.9) |
| Perception of Nepal Dental Association, N (%) | | |  |  |  |
|  | Had appropriate support | 8 (14.6) | 14 (16.3) | 14 (25.5) | 28 (31.1) |
|  | Did not have appropriate support | 47 (85.5) | 72 (83.7) | 41 (74.6) | 62 (68.9) |

1. The total number of dentists in each question may not have been 58 for financial, 95 for material, 61 for technical, and 105 for guideline/guidance because the analysis excluded those who answered “refuse to answer”.
